# Feel It, See It, Get It: Is Internal Medicine Residents’ Use of Ultrasound in Lumbar Puncture Associated With Higher Success Rate?

**DOI:** 10.7759/cureus.11545

**Published:** 2020-11-18

**Authors:** Mohammed G Elhassan, Mossab Mohameden, May Kam, April Manalaysay, Ratnali V Jain

**Affiliations:** 1 Internal Medicine, Saint Agnes Medical Center, Fresno, USA; 2 Rheumatology, University of Cincinnati Health, Cincinnati, USA; 3 Hospital Medicine, Chest and Critical Care Consultants, Los Angeles, USA; 4 Hospital Medicine, Kaweah Delta Medical Center, Visalia, USA; 5 Clinical Research Center, University of California San Francisco Fresno, Fresno, USA

**Keywords:** internal medicine residents, lumbar puncture, ultrasound, medical procedures, medical education

## Abstract

Introduction: Multiple emergency medicine and anesthesiology research studies suggest that ultrasound (US) is potentially useful in assisting with needle insertion in a lumbar puncture (LP). However, little is known about its value when utilized by internal medicine (IM) residents. The objective of this study is to examine whether the use of ultrasound in LPs performed by internal medicine residents is associated with a higher success rate than the traditional palpation method.

Materials and Methods: We reviewed all LP procedure notes in our hospital's records written by IM residents from June 2017 to December 2018 in a single community teaching hospital. We examined the association between the US use and success using the Chi-squared test and logistic regression model.

Results: Among the 152 lumbar punctures documented, 130 specified whether US was used or not. Among these, 39 were ultrasound-assisted and 91 were not. Use of ultrasound was associated with a higher success rate compared to the non-ultrasound-use (87% vs 73%; p=0.1). The association was strengthened using logistic regression but did not reach statistical significance (OR 3.5; CI: 0.9 -13.8; p=0.07). Success was significantly associated with a fewer number of attempts (p<0.001). No statistically significant association was found between success and patients’ body mass index (BMI; p=0.57), or level of training (p=0.11).

Conclusions: Use of ultrasound for needle insertion in lumbar punctures performed by internal medicine residents was associated with a higher success rate compared to the palpation method but without statistical significance. Ultrasound is a non-invasive, quick, and safe tool. Our study favors its use as an aid during lumbar puncture when performed by internal medicine residents. Larger studies are needed to gather more evidence in support of this conclusion.

## Introduction

Lumbar puncture (LP) is a very important diagnostic and therapeutic procedure commonly ordered and performed by internal medicine (IM) residents [1,2]. Knowledge and cognitive competence related to this medical procedure count toward the graduation requirements for IM residency programs, although competence to perform the procedure is not an absolute requirement. Nevertheless, the American Board of Internal Medicine (ABIM) believes that “residents should be active participants in performing procedures,” including LPs [3].

Conventionally, LPs are performed with the palpation method, where the operator palpates the anatomic landmarks around lumbar spines (L) to identify the needle insertion site (usually L3/L4 or L4/5 interspinous spaces). This can be difficult in certain patients (e.g., obese or pregnant patients). Ultrasound (US) can be a safe, non-invasive, easy to learn, and cost-effective tool that can assist in identifying the anatomic landmarks [4-8]. The first use of US for this purpose was described by Russian anesthesiologists in 1971 [9]. Since then, several studies have been done to compare the success and complication rates of LPs done with and without US use [4,10-16]. Of these studies, only one study [10] was able to achieve double-blinded randomization using ultraviolet ink markings visible only under ultraviolet light. This study on LPs done by emergency medicine residents showed a significantly higher success rate of LPs done with US assistance to mark the needle insertion site. Nevertheless, other clinical trials showed no significant difference between LPs done with and without US assistance [7,14,17,18].

Most of the studies we found in the literature were done in emergency or anesthesia settings. To the best of our knowledge, no studies evaluated IM residents' use of US to assist with LPs and little is known about whether US increases the rate of successful LPs when performed by this group of learners. We hypothesize that the use of US will likely help IM residents achieve higher LP success rates since it can directly visualize the appropriate interspinous space, thus allowing for better insertion site marking. It can also help identify patients with narrower spaces who may need a referral for fluoroscopy-guided LP by interventional radiology.

In this study, we aimed to retrospectively examine the success rate of LP performed by IM residents in one IM program when US is used to mark the insertion site and compare that to the success rate when only the palpation method is used.

## Materials and methods

We did a retrospective review of electronic health records on all LP procedures performed by IM residents at all the three training levels on adult patients (16 years of age and older) who were admitted to the general medical wards or intensive care unit in a tertiary community teaching hospital during the period between June 1, 2017, and December 10, 2018. The following data were collected from the procedure note as documented by the operator: whether US was used to mark needle insertion site or not, the number of attempts, procedure success (defined by obtaining cerebrospinal fluid [CSF] for testing or therapeutic purposes), patient’s body mass index (BMI), and resident’s postgraduate year (PGY) of training. Descriptive analyses included frequencies and percentages for categorical data, and a two-tailed Chi-Squared test was used to compare proportions. A logistic regression model was used to examine the association between the success of LP and other variables namely: US use, age, gender, BMI, level of training, and the number of attempts. Statistical analysis was performed using IBM SPSS Statistics, Version 25 (IBM Corp., Armonk, NY), and a two-tailed p-value of <0.05 was used to determine statistical significance. This study was approved by the institutional review board of the facility.

## Results

There were 152 LPs documented by IM residents during our study period. We excluded 22 procedures due to missing documentation whether US was used to mark the needle insertion site or not. In the remaining 130 LPs, 39 were done with the use of US, and 91 were performed without US. Characteristics of some variables between the US-assisted and the non-US-assisted groups are shown in Table 1.

**Table 1:** Characteristics of the ultrasound-assisted and the non-ultrasound-assisted lumbar puncture groups. BMI: body mass index, LP: lumbar puncture, PGY: postgraduate year, CSF: cerebrospinal fluid.

Variable	US-assisted LP group (n=39)	Non-US-assisted LP group (n=91)	p-Value
Mean age of patients in years	41	47	0.05
Gender of patients: male; n (%)	30 (77%)	64 (70%)	0.41
Mean BMI of patients	28.3	26.7	0.18
PGY-level of the operator, n (%)			
PGY-1	28 (72%)	49 (54%)	0.17
PGY-2	9 (23%)	27 (30%)	0.41
PGY-3	2 (5%)	15 (16%)	0.01
Number of attempts; mean	2.4	2.2	0.44
Successful (CSF obtained); n (%)	34 (87%)	67 (73%)	0.10

**Figure 1:**
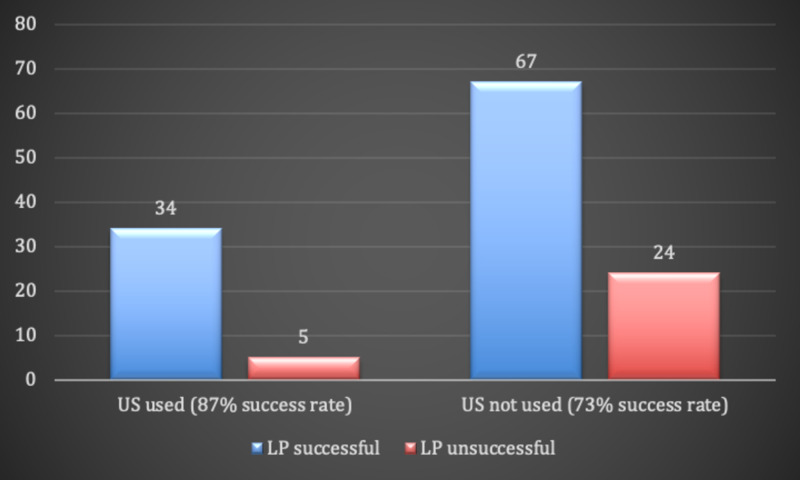
Number of successful and unsuccessful LP procedures in the US-assisted and non-US-assisted groups. LP: lumbar puncture, US: ultrasound.

More than two-thirds of US-assisted LPs were done by the least experienced residents (PGY-1 residents) compared to about half for non-US-assisted LPs. Nevertheless, US-assisted LPs were associated with a higher success rate compared to non-US-assisted LPs, but the difference did not reach statistical significance (87% vs 73%, respectively, p=0.1; Figure 1).

Using the logistic regression model for controlling BMI level or training of the operating resident, successful LPs were more likely to be performed by US, but again this was not statistically significant (odds ratio of 3.5 [CI: 0.89-13.8]; p=0.07; Table 2).

**Table 2:** P-values and OR for successful lumbar puncture procedures using the logistic regression model. BMI: body mass index, PGY: postgraduate year, OR: odds ratio, CI: confidence interval.

Variable	p-Value	Adjusted OR	95% CI	
			Lower	Upper
Age of patients	0.745	1.006	0.972	1.040
Gender of patients	0.544	1.427	0.453	4.491
BMI of patients	0.571	1.022	0.947	1.104
PGY level of operator	0.110	0.507	0.221	1.165
Ultrasound use	0.073	3.508	0.891	13.817
Number of attempts	<0.001	3.070	1.991	4.734

There was a statistically significant association between success and lower number of attempts (odd ratio of 3.07 [CI: 1.9-4.7]; p <0.001), but no statistically significant association was found between success and patients’ BMI (p=0.57), or level of training (p= 0.11).

## Discussion

Although several studies were have been published to evaluate LP success with US assistance, most of these studies were done in the emergency department or by radiologists or anesthesiologists. To the best of our knowledge, this is the first study to evaluate the success rate of LP procedures done by IM residents with and without US assistance. Two-thirds of operators in the US group were the least experienced residents (PGY-1) compared to about half in the non-US group. Nevertheless, the success rate was higher with the US use compared to the non-US use and this association was further strengthened when a logistic regression modelling was used to account for other variables. However, the difference between the two study groups did not reach statistical significance. This finding is likely because of the small sample size and will need further investigations using a larger sample size to strengthen the association. The smaller number of LPs done with the help of US in our program is likely due to the fact that most faculty and residents are traditionally trained to use the palpation method to mark the needle insertion site, with only a few faculty attendings who are trained on the use of US for that purpose. This trend of using US for LP in our teaching service did not start until about a few months before the start time of this study. Following the American Board of Internal Medicine's (ABIM's) guidelines encouraging residents to perform procedures, this residency program greatly supports the residents’ training on the common bedside procedures that are usually performed by IM physicians, including LPs.

Compared to other bedside medical procedures (e.g., central lines, thoracentesis, and paracentesis), there are some conflicting data on the use of US for LP. One major study that supported the use of US for spinal procedures (LPs and epidural catheterization) is a systematic review and meta-analysis of 14 clinical trials [11]. It examined whether US reduces the risk of failed spinal procedures compared to the standard palpation method. This meta-analysis also looked at whether the use of US can reduce the risk of traumatic procedures, insertion attempts, and needle redirections, and it was found that the use of US reduced the risk of failed procedures compared to standard palpation methods. The number of traumatic procedures was also found to be reduced when the US is used. Similarly, the number of insertion attempts was reduced with the US use as well as the number of needle redirection. One the other hand, a prospective study that examined the use of US in LP by emergency medicine residents did not find a statistically significant difference in the success rate between the two groups, although a statistically significant difference in the number of attempts was shown in favor of the US group [6]. 

**Figure 2:**
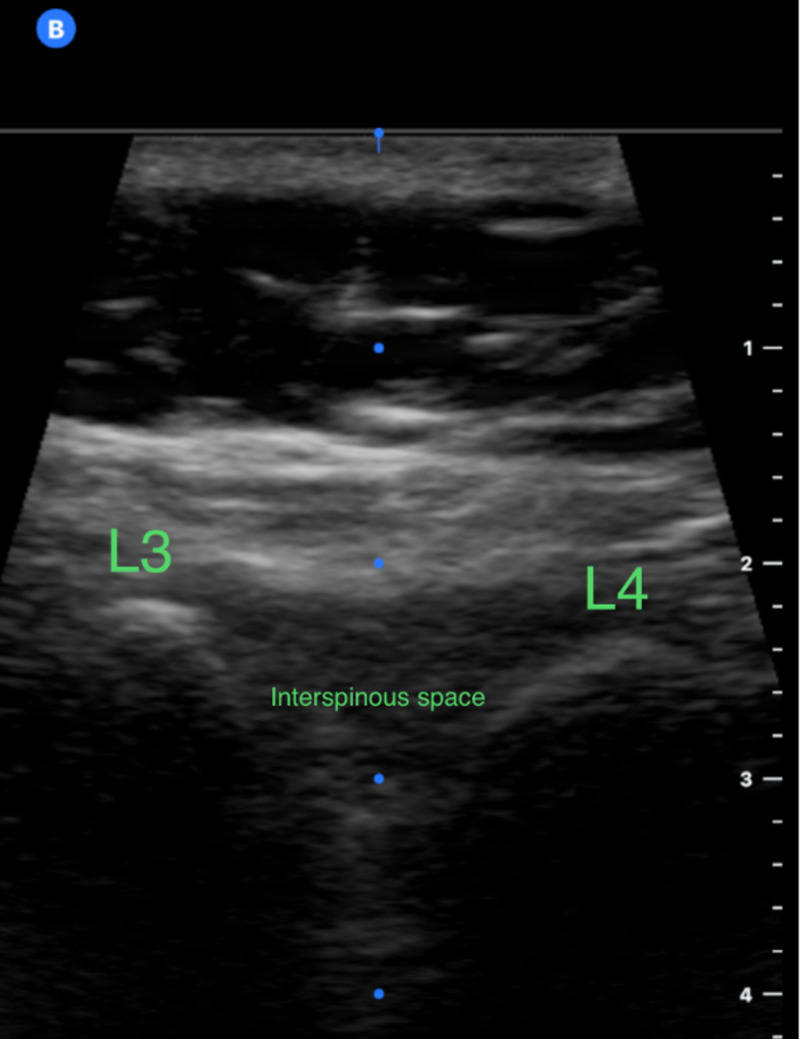
Ultrasound image of lower lumbar spines (L) using high-frequency probe showing the L3-L4 interspinous space suitable for spinal needle insertion for lumbar puncture (L3 and L4 spinous processes appears as hyperechoic [white] curved lines).

Palpation of landmarks for LPs can be difficult particularly in obese patients [19]. One study looked at the rate of successful palpation in patients with varying BMIs and found that the higher the BMI was associated with lower the chance of successful identification of pertinent landmarks for LP [4]. The use of US was found to increase the chance of successful palpation of these landmarks in patients with high BMI. This study also evaluated the distance between the skin and ligamentum flavum using US in patients with normal BMI compared to patients with BMI > 30 and found that the difference can be up to 2 cm. Thus, US can be very helpful in determining this distance and therefore choosing and using the appropriate needle size. US can also help identify the widest interspinous space thus increasing the LP success rate. The standard palpation method is inferior and less accurate in identifying the lumbar spaces when compared to the US-guided method [8]. The use of US can also make the identification of lumbar space visualized more clearly since the operator can see the space directly (Figure 2).

Our study results support the notion that the US use can help increase LP success rate when done by IM residents, although it was not powered enough to reach statistical significance. With the expanding role of bedside US for medical procedures and also for clinical diagnostic purposes, many medical schools and residency programs now train their learners on the use of bedside US [20,21]. Medical students and residents are becoming more familiar with the different clinical utilities of US than ever before, given its various advantages over other radiological tools (safe, relatively cheap, quick to perform, and can be available at the bedside). 

Our study has several limitations. Data collection was done retrospectively and so it carries the inherent drawbacks of retrospective studies, including the inability to control for confounding factors that might affect LP success (e.g., patient positioning, prior number of LPs performed by each operator). Also, some procedure notes lacked the documentation of US use and thus decreased the power of the study. Also, it was conducted in a single residency program. US is operator dependent and any study evaluating the US use will likely be limited by operators’ factors, including experience, and also by the experience of supervising faculty. Most of the LPs were done by PGY-1 residents who have relatively less experience with US use in general. However, we still observed a trend toward more LP success with US and this might reflect its usefulness for novice learners. It is likely that different US machines were used by residents depending on the location of the patient, but this is unlikely to be a major issue as the image needed to locate the interspinous space is standard in terms of the anatomy of the area. Finally, the number of LP attempts is likely to be underestimated by residents when documented in notes and some may not consider needle re-direction as an attempt. Nevertheless, we think our results open a door for further studies to examine how the US can assist medical residents during LP procedures and whether its use can be significantly associated with more success rate in larger prospective studies. In addition, other factors like time to complete the procedure, patient pain experience, and/or traumatic tap, can also be included in future studies. 

## Conclusions

Ultrasound-guided lumbar puncture performed by internal medicine residents was associated with a higher rate of success, even when performed by PGY-1 residents who usually have the least clinical experience, even though this was not statistically significant. Procedure success was also associated with fewer attempts. Additional prospective studies with a larger sample size are needed to closely examine this association further.
